# Variants in the *VDR* Gene May Influence 25(OH)D Levels in Type 1 Diabetes Mellitus in a Brazilian Population

**DOI:** 10.3390/nu14051010

**Published:** 2022-02-27

**Authors:** Rafaella S. Ferraz, Caio S. Silva, Giovanna C. Cavalcante, Natércia N. M. de Queiroz, Karem M. Felício, João S. Felício, Ândrea Ribeiro-dos-Santos

**Affiliations:** 1 Laboratory of Human and Medical Genetics, Federal University of Para, Belem 66075-110, PA, Brazil; rafaellaferraz.16@hotmail.com (R.S.F.); scaio@hotmail.com (C.S.S.); giovannaccavalcante@gmail.com (G.C.C.); 2 Endocrinology Research Center, Joao de Barros Barreto University Hospital, Federal University of Para, Belem 66075-110, PA, Brazil; natercianeves@hotmail.com (N.N.M.d.Q.); karemfelicio@yahoo.com.br (K.M.F.); felicio.bel@terra.com.br (J.S.F.); 3 Oncology Research Center, Joao de Barros Barreto University Hospital, Federal University of Para, Belem 66073-000, PA, Brazil

**Keywords:** vitamin D receptor, 25(OH)D, genetic polymorphism, genomic ancestry, Type 1 diabetes

## Abstract

Vitamin D has been considered a strong contributing factor to type 1 diabetes mellitus (T1DM). Many studies have investigated polymorphisms in the *VDR* gene in association with T1DM in different populations, but there are still conflicting findings. This study aimed to evaluate the association of four variants in the *VDR* gene (rs7975232, rs1544410, rs731236, and rs2228570) with T1DM risk and vitamin D levels within a population from North Region, Brazil, as well as the influence of genomic ancestry on T1DM. A total of 65 T1DM patients and 83 non-T1DM patients were enrolled in this study. *VDR* gene polymorphisms were assessed using Sanger sequencing analysis. Genomic ancestry was analyzed using a set of 61 ancestry-informative markers. T1DM patients showed higher European genomic contribution and lower Native American genomic contribution when compared to non-T1DM patients. T1DM patients with AA genotype in rs1544410 or CC genotype in rs731236 had significantly lower 25(OH)D levels compared to the other two genotypes (*p* = 0.013 and *p* = 0.02, respectively), while T1DM with TT genotype in rs2228570 had higher 25(OH)D levels compared to CC + TC in the same polymorphism (*p* = 0.011). Our findings suggest that the association between 25(OH)D and T1DM may be modified by *VDR* variants, possibly influencing the development of this autoimmune disease.

## 1. Introduction

Type 1 diabetes mellitus (T1DM) is an autoimmune disease caused by complex interactions between immunological, genetic, epigenetic, and environmental factors, leading to the destruction of pancreatic β-cells and subsequent insulin deficiency [1]. T1DM affects any age group, although the onset occurs most frequently in children and adolescents. Globally, its prevalence is around 1.1 million individuals and this figure has been rising by 3% annually. Notably, Brazil has the third largest number of children and adolescents with T1DM in the world [2].

In recent years, vitamin D has been considered an important factor to T1DM [3], given the high prevalence of vitamin D deficiency in T1DM patients compared to non-diabetic subjects and its impact on glycemic control [4]. Moreover, vitamin D has many immunomodulatory effects, including increased infiltration of regulatory T cells, inhibition of Th1 infiltration, and decreased expression of inflammatory chemokines and cytokines (e.g., IL-1β, IP-10, IL-15). Together, these effects lead to reduced severity of insulitis and preserved β-cell functionality, inhibiting the development of T1DM [5,6,7]. In addition, vitamin D deficiency leads to impaired secretion of insulin [8] and higher risk of islet autoimmunity [9].

These biological actions of vitamin D are mediated by the vitamin D receptor (VDR), protein encoded by the *VDR* gene [10]. This gene is located at 12q13 and contains four polymorphism sites extensively studied in association with the disease: rs7975232 (*ApaI*, G > T) and rs1544410 (*BsmI*, G > A), both present in intron 8; rs731236 (*TaqI*, T > C) present in exon 9; and rs2228570 (*FokI*, T > C) present in exon 2 [11]. Variants rs7975232, rs1544410, and rs731236 are located near the 3′ end of the *VDR* gene and are likely associated with changes in mRNA stability or are in linkage disequilibrium (LD) with nearby functional variants [12]. In contrast, variant rs2228570 causes a change in the translation start site and results in a shorter protein [13].

Many studies have investigated these polymorphisms in the *VDR* gene in association with T1DM within different populations [14,15]. However, besides resulting in conflicting findings, these studies are carried out in other countries and do not represent the highly admixed Brazilian population and/or do not consider the population substructure effect (i.e., variation in the proportions of genomic ancestry among subpopulations) [16].

Brazil has one of the most heterogenous genetic patterns in the world [17]. It has an admixed population mainly based in three ancestries (European, Native American, and African), receiving genomic influence from all of them [16]. Considering that the admixture process may distort LD patterns and combine allele frequency distribution from distinct parental populations [18], allelic frequency studies in this population may be performed to find new associations and to identify alleles shared with other populations, which help to understand susceptibility patterns in diseases. Therefore, the aim of the present study is to investigate the association of rs7975232, rs1544410, rs731236, and rs2228570 with T1DM risk and vitamin D levels in a population from North Region, Brazil, as well as the influence of genomic ancestry on the development of T1DM.

## 2. Materials and Methods

### 2.1. Ethical Approval

This study was approved by the Research Ethics Committee of João de Barros Barreto University Hospital (HUJBB, Belem, Para, Brazil) (number 0122.0.071.000-12), and all participants gave written informed consent. All procedures performed in the present study involving human participants were conducted according to the ethical guidelines of the Declaration of Helsinki.

### 2.2. Subjects

This case–control study included 65 T1DM patients, diagnosed according to the World Health Organization and the American Diabetes Association (ADA) [19,20], and 83 unrelated individuals with no history of T1DM (herein after called “non-T1DM”) and with normal blood glucose levels. All subjects were recruited from the Endocrinology and Metabology/Diabetes Unit of the Joao de Barros Barreto University Hospital at the Federal University of Para (HUJBB-UFPA). We collected 5 mL of peripheral blood samples with EDTA from all participants before any treatments or supplementations. Individuals that already supplemented vitamin D or had been diagnosed with chronic illnesses that alter vitamin D metabolism were not included in this study.

### 2.3. Biochemical Assays

The metabolite used to assess vitamin D status was 25-hydroxivitamin D (25(OH)D). Vitamin D assays were performed using the appropriate protocols for chemiluminescent microparticle immunoassay (CMIA) in an external laboratory, Amaral Costa Laboratory (Belem, Brazil). Reference ranges of vitamin D (25(OH)D) were established according to the Endocrine Society Clinical Practice Guidelines, in which levels below 30 ng/mL were considered a vitamin D insufficiency and levels above 30 ng/mL were considered vitamin D sufficiency [21].

### 2.4. DNA Extraction and Quantification

Nuclear DNA was extracted from EDTA whole blood using a phenol-chloroform method, based on Sambrook et al. (1989) [22]. DNA quantification was performed with a NanoDrop 1000 spectrophotometer (Thermo Fisher Scientific Inc., Wilmington, DE, USA).

### 2.5. Genotyping

The DNA was amplified by polymerase chain reaction (PCR) using specific primers for rs7975232, rs1544410, rs731236, and rs2228570. Primer sequence descriptions of the four single nucleotide polymorphisms (SNPs) are shown in Table S1. The PCR reaction system included 0.5 µL primer, 2.5 µL PCR buffer, 0.75 µL MgCl_2_, 2.0 µL dNTP, 0.2 µL Taq polymerase, 1.0 µL DNA, and 17.55 µL water to a final reaction volume of 25 µL. PCR was carried out at Thermo Fisher Scientific Veriti^TM^ Thermal Cycler(Thermo Fisher Scientific Inc., Wilmington, DE, USA) using the following procedures: 1 cycle of 95 °C denaturation for 10 min, 35 cycles of 95 °C denaturation for 15 s, annealing temperature adjusted by primer for 30 s, and 72 °C extension for 1 min 30 s. Thereafter, this PCR product went through sequencing reaction using BigDye^TM^ Terminator v3.1 Cycle Sequencing Kit (Thermo Fisher Scientific Inc., Wilmington, DE, USA) in ABI PRISM 3130 Genetic Analyzer (Thermo Fisher Scientific Inc., Wilmington, DE, USA). The produced nucleotide sequences were analyzed with the Sequencing Analysis Software v5.2 (Thermo Fisher Scientific Inc., Wilmington, DE, USA).

### 2.6. Analysis of Genomic Ancestry

Due to this population formation with multiple genomic ancestry contributions, Brazil is affected by the substructure effect, which in association studies can confer spurious results [16]. Furthermore, genomic ancestry–environmental interaction may play a role in multifactorial diseases, including T1DM [23]. Therefore, the studied samples were analyzed using a previously developed set of 61 ancestry-informative markers (AIM), following the established protocols [16,23]. The proportions of European, African, and Native American ancestries were estimated using Structure v.2.3.4 [24].

### 2.7. Statistical Analyses

All statistical analyses and plotting were performed with R v2.14.1 [25]. Hardy–Weinberg Equilibrium (HWE), Linkage Disequilibrium, and allelic and genotype frequencies were performed using a SNPassoc package [26]. A Haplotype test was performed using a Haplo.Stats package [27]. *F_ST_* was calculated using Weir and Cockerham (1984) equations [28] with a Hierfstat package [29]. The categorical variables were assessed by a Chi-squared test. A Mann–Whitney test was used to obtain ancestry estimates. A T-test and ANOVA were used to compare 25(OH)D levels with genotype frequencies. Benjamini–Hochberg (FDR) correction for multiples comparisons was applied when necessary. The *p*-value was considered statistically significant when lower than 0.05.

## 3. Results

### 3.1. Characteristics of the Subjects

A total of 65 T1DM and 83 non-T1DM individuals were included in the study. The mean age at diagnosis of the T1DM group was 11.67 ± 7.94 years old. Statistically significant differences were detected in the sex, age, weight, and body mass index (BMI) comparisons between the groups, as shown in Table 1. At baseline 25(OH)D levels, T1DM had significantly lower levels when compared to non-T1DM (26.04 ± 8.45 ng/mL vs. 32.60 ± 8.85 ng/mL, *p* = 5.832 ×10^−6^) (Table 1).

**Figure 1:**
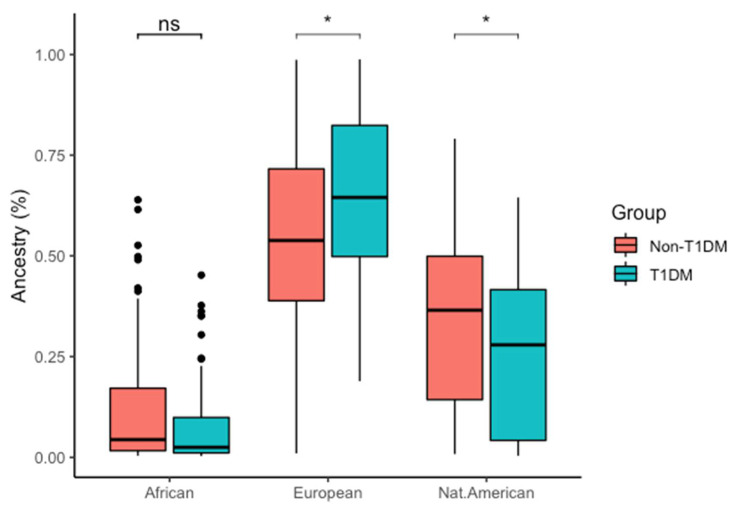
Genomic ancestry comparison between T1DM and non-T1DM groups. ns: *p* > 0.05, * *p* < 0.05.

Different ancestry contribution was estimated for both groups. T1DM showed 0.08 ± 0.11 African, 0.65 ± 0.22 European and 0.26 ± 0.2 Native American, while non-T1DM showed 0.12 ± 0.16 African, 0.54 ± 0.24 European, and 0.34 ± 0.21 Native American contributions. T1DM showed higher European genomic ancestry contribution when compared to non-T1DM, while non-T1DM showed higher Native American contribution than T1DM (Figure 1 and Figure S1).

### 3.2. VDR Genotype and Allele Distributions and Risk of Type 1 Diabetes

Due to insufficient sample quantity, it was not possible to genotype rs1544410 in non-T1DM. The genotype frequencies of all SNPs (rs7975232, rs1544410, rs731236, and rs2228570) were in HWE in both T1DM and non-T1DM. Polymorphisms rs7975232, rs1544410, and rs731236 showed strong LD among them, while rs2228570 showed weak LD with all the others (Table S2). No association between the investigated *VDR* polymorphisms and risk of T1DM was found in our population, except in the overdominant model for rs731236 (Tables S3–S5). In the haplotype test, there was also no association between the haplotype block and T1DM (Table S6).

**Figure 2:**
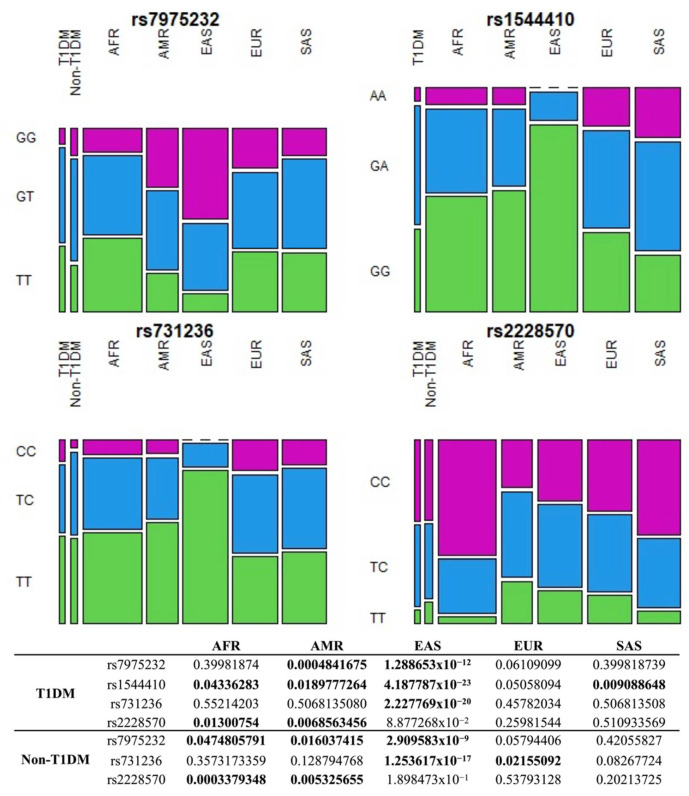
Pairwise comparison of genotype frequencies of *VDR* polymorphisms in T1DM and non-T1DM with each of the five continental populations from the 1000 Genomes database. Values in bold indicate a statistically significant difference (*p*-value < 0.05). AFR: African, AMR: American, EAS: East Asian, EUR: European, SAS: South Asian, T1DM: Type 1 diabetes mellitus.

**Figure 3:**
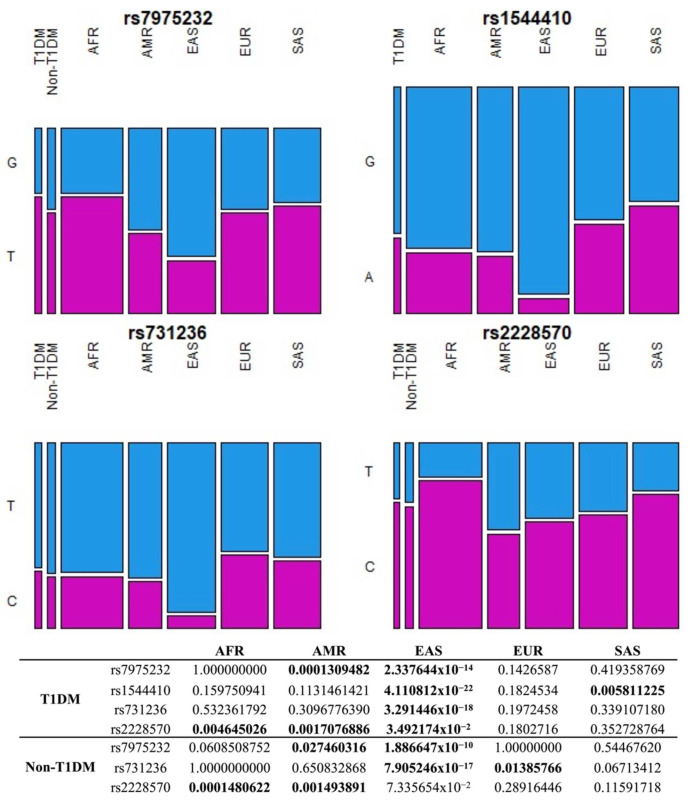
Pairwise comparison of allele frequencies of *VDR* polymorphism in T1DM and non-T1DM with each of the five continental populations from the 1000 Genomes database. Values in bold indicate a statistically significant difference (*p*-value < 0.05). AFR: African, AMR: American, EAS: East Asian, EUR: European, SAS: South Asian, T1DM: Type 1 diabetes mellitus.

In addition, pairwise comparisons of allelic and genotypic frequencies of *VDR* polymorphisms in T1DM and non-T1DM with each one of the five continental populations (African, American, East Asian, European, and South Asian) from the 1000 Genomes database [30] were performed. Results demonstrate that frequencies of rs7975232, rs1544410, rs731236 and rs2228570 in T1DM do not differ from those in Europeans, while the frequencies of rs7975232, rs731236, and rs2228570 in non-T1DM do not differ from South Asians. Both groups have the most discrepant frequencies from East Asians (Figure 2 and Figure 3). Indeed, East Asians are the population that most differed from T1DM and non-T1DM in inter-populational variability of the variants assessed by the fixation index (*F_ST_*), while African, South Asian, and European populations were the most similar (Figure S2).

### 3.3. VDR Genotypes and 25(OH)D Levels

**Figure 4:**
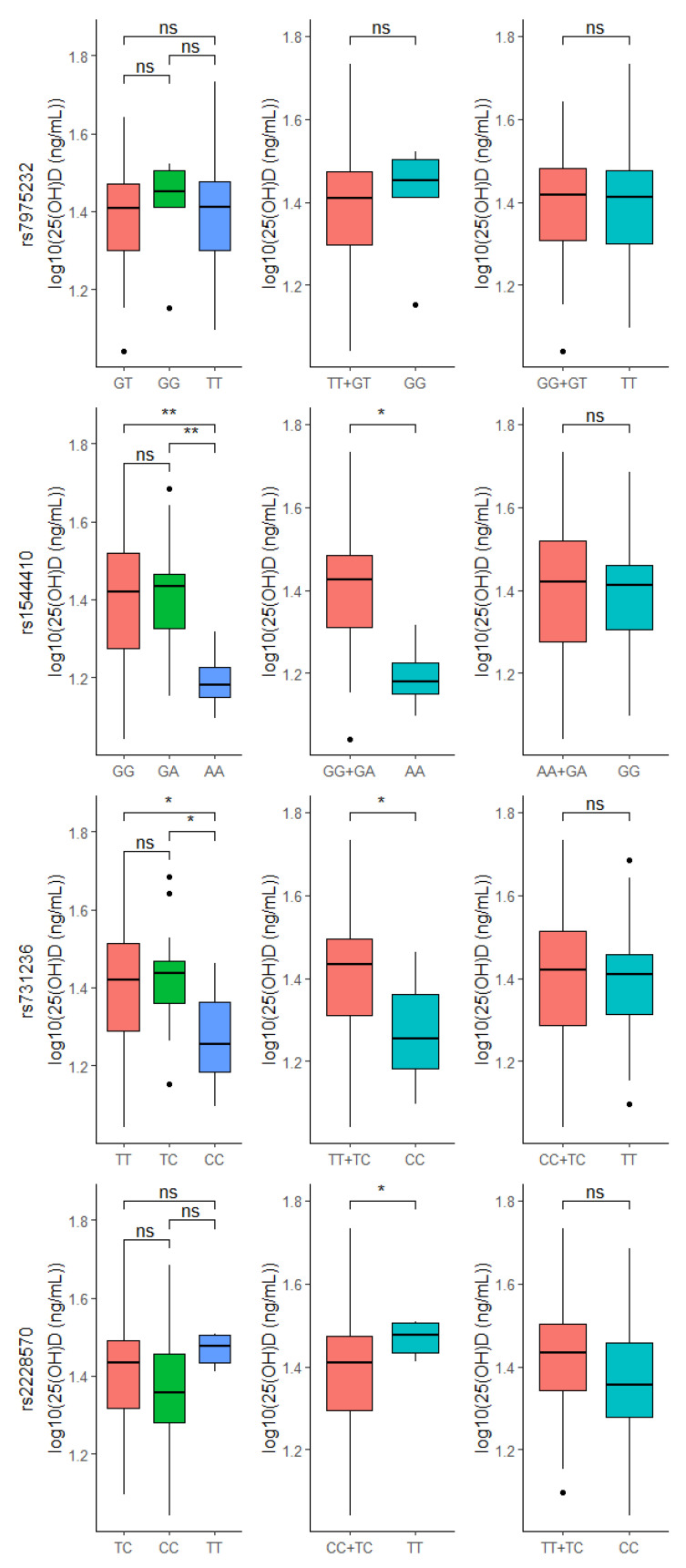
Comparison between 25(OH)D levels and *VDR* genotypes within T1DM group. ns: *p* > 0.05, * *p* < 0.05, ** *p* < 0.01.

Importantly, significant differences between 25(OH)D levels and *VDR* genotypes were found. T1DM individuals with AA genotype in rs1544410 or CC genotype in rs731236 had lower 25(OH)D levels compared to the other two genotypes, while T1DM with TT genotype in rs2228570 had higher 25(OH)D levels compared to CC + TC in the same polymorphism (Figure 4). Interestingly, differences between 25(OH)D levels and *VDR* genotypes were not found in non-T1DM (Figure S3).

## 4. Discussion

Over the last few decades, many studies have explored the effects of vitamin D in the immune system. Given the possible role that vitamin D plays in autoimmune diseases, its levels have been investigated in T1DM patients [31]. In the present study, T1DM patients had significantly lower 25(OH)D levels when compared to non-T1DM patients. In addition, 75% of T1DM patients showed vitamin D insufficiency. These findings reinforce the link between vitamin D insufficiency and T1DM reported in previous studies [32,33,34,35].

Vitamin D is known to play a protective role in T1DM through the *VDR* expressed in pancreatic β-cells, activated T cells, and antigen presenting cells [33]. These findings led to the investigation of *VDR* gene polymorphism in association with T1DM in different populations [36]. In our cohort, no association between rs7975232, rs731236, or rs2228570 and T1DM was detected. Similar results have been reported in populations from Portugal [14], the United Kingdom [37], Finland [37], Norway [37], Romania [37], and the United States [37]. However, positive associations have been found in Kuwaiti [38], Saudi [39], Pakistani [40], Japanese [41], and Korean populations [15].

The reasons for these discrepant results might be different allelic and genotypic distribution between the populations, as well as environment–gene interactions in the development of T1DM [36,42]. Indeed, in the meta-analyses by Zhai et al. (2020) [43] and Tizaoui et al. (2014) [44] there was no statistical evidence of overall association between *VDR* polymorphisms and T1DM, but when these analyses were subdivided by ethnicity, the association was found.

Notably, the Brazilian population is one of the most genetically diverse worldwide, as it results from colonization processes of different ancestral populations. Native Americans were the first settlers in the territory that would become Brazil, followed by the migration of Europeans, mainly Portuguese, starting in 1500. Lastly, from the second half of the 16th century to 1850, there was an intense process of slave trading originating from different African countries [17]. Thus, the Brazilian population is formed by the admixture of genetic contributions of three main ancestral populations—Europeans, Africans, and Native Americans [16]. In this study, conducted in the North Region of this country, T1DM had greater European genomic ancestry and lower percentage of Native American contribution in comparison to non-T1DM. In a previous study, similar results were reported in all geographical regions of Brazil (Southeast, Midwest, Northeast, North and South), where T1DM patients presented a higher percentage of European ancestry and lower Native American contribution compared to non-T1DM Brazilian individuals [45]. In general, the incidence of T1DM is believed to be higher in countries in Europe than in other continents [46]. Together, these findings suggest an influence of European genomic ancestry in the development of T1DM.

To clarify the *VDR* variants distribution in admixed Brazilians and correlate this variability with genomic ancestry, we performed pairwise comparison of these polymorphisms in T1DM and non-T1DM with each of the five continental populations from the 1000 Genomes database. In the pairwise comparisons between T1DM and Europeans, there were no differences in allelic and genotypic frequencies of *VDR* polymorphisms, highlighting the similarity of the studied group with the European population. However, one of these polymorphisms (rs2228570) has been previously associated with decreased risk of T1DM in Europeans [43], but not in our population.

This same polymorphism was reported in association with increased risk of T1DM in Africans [43]. In a pairwise comparison between T1DM and non-T1DM with Africans, significant allelic difference was reported, showing genetic distinction and subsequent difference in susceptibility to T1DM between Africans and our population. Of all continental populations from 1000 Genomes, East Asians were the most distinct from the Brazilian population, while Africans, South Asians, and Europeans were the most similar. Allele frequencies similarity between our population and European and African populations are expected considering that, historically, Brazil is genetically constituted by these populations. This analysis reinforces the contribution of different genomic ancestries in our admixed population and would help to explain the *VDR* association with T1DM. However, there was no association of the studied polymorphisms with T1DM, probably due to the limited sample size.

As for vitamin D levels, significant differences between 25(OH)D levels and *VDR* genotypes were found in T1DM. The 25(OH)D levels of patients with AA (rs1544410) or CC (rs731236) genotypes were significantly lower than in individuals carrying other genotypes for these polymorphisms. Similarly, Cobayashi et al. (2015) [47] reported that Brazilian Amazonian children with A allele in rs1544410 had increased risk of low serum 25(OH)D and this allele was positively associated with glucose concentration and HOMA-IR (i.e., parameter that measures insulin resistance). Recently, a study in Northeast Region, Brazil, reaffirmed the link between AA (rs1544410) and high glycemic levels [48].

Curiously, in our study, T1DM patients with TT (rs2228570) genotype had high levels of 25(OH)D, suggesting a protective effect of this genotype. However, Morán-Auth et al. (2015) [49] demonstrated an association of this genotype with an increase in the percentage of T CD4^+^ cells (T1DM driver cells) under vitamin D stimulation in T1DM, demonstrating a possible risk effect of this genotype for the disease.

In the present study, no statistically significant differences were found between the investigated variants and 25(OH)D levels in the control group, so that all significant results in the analyses were found only in T1DM patients. This suggests that the association between 25(OH)D and T1DM may be modified by *VDR* variants, in which possibly AA (rs1544410) and CC (rs731236) are associated with lower vitamin D levels and indirectly with the risk of T1DM, while TT (rs2228570) is related to high levels of 25(OH)D. Unfortunately, the sample collection had to be interrupted at the beginning of the COVID-19 pandemic. We acknowledge that it is a small sample size, however, even so, we obtained interesting and promising results on the influence of *VDR* genotypes on vitamin D levels in T1DM. Therefore, we encourage further studies combining measurement of metabolic parameters and *VDR* polymorphisms in larger cohorts to strengthen our findings and clarify the effect of vitamin D on T1DM patients with the relevant genotypes.

## 5. Conclusions

In summary, although variants rs7975232, rs731236, and rs2228570 do not seem to play a direct role in the susceptibility to T1DM within the studied population, our results demonstrate a significant association of the rs1544410, rs731236, and rs2228570 variants with 25(OH)D levels in T1DM, which may influence the development of this disease. In addition, corroborating previous studies, our genomic ancestry results suggest that a higher European ancestry contribution is associated with a higher risk of T1DM.

## Figures and Tables

**Table 1:** Clinical characteristics of T1DM and non-T1DM groups.

	T1DM (*n* = 65)	Non-T1DM (*n* = 83)	t/χ 2	*p*-Value
Sex				
Female	35 (53.85%)	64 (77.10%)	7.8875	0.004978 *
Male	30 (46.15%)	19 (22.90%)		
Age (year) ^1^	27.28 ± 10.38	38.49 ± 13.55	−5.5488 ^3^	1.452 × 10^−7^ *
Diagnosis age (year) ^2^	11.67 ± 7.94	-		
Weight (kg)	62.03 ± 14.48	70.06 ± 15.88	−3.2969 ^3^	0.00125 *
BMI (kg/m^2^)	23.85 ± 4.23	27.64 ± 5.12	−4.9397	2.121 × 10^−6^ *
25(OH)D (ng/mL) ^4^				
(0, 30]	48 (75%)	34 (41.46%)	14.904	0.0001027 *
(30, ∞]	16 (25%)	48 (58.54%)		
25(OH)D (ng/mL)	26.04 ± 8.45	32.60 ± 8.85	−4.7382 ^3^	5.832 × 10^−6^ *

* Statistically significant result, *p* < 0.05. ^1^ Refers to the age at which the participant was enrolled in this study, ^2^ Refers to the age at which the participant was diagnosed, ^3^ *t*-test performed with values in log10, ^4^ The information on 25(OH)D was missing for one individual with T1DM and one non-T1DM. Abbreviations: BMI, body mass index; T1DM, type 1 diabetes mellitus; 25(OH)D, 25-hydroxyvitamin D.

## Data Availability

The data presented in this study are available on request from the corresponding author. The data are not publicly available due to data privacy.

## Supplementary Materials

The following supporting information can be downloaded at: https://www.mdpi.com/article/10.3390/nu14051010/s1, Figure S1: Distribution of African, European, and Native American genomic ancestry in T1DM and non-T1DM. Each symbol represents an individual and its position on the graph is related to the proportion of the corresponding ancestry; Figure S2: Heatmap of pairwise *F_ST_* between T1DM and non-T1DM and each of the five continental populations from 1000 Genomes database. AFR: African, AMR: American, EAS: East Asian, EUR: European, SAS: South Asian, T1DM: type 1 diabetes mellitus; Figure S3: Comparison between 25(OH)D levels and *VDR* genotypes within non-T1DM group. ns: *p* > 0.05. Table S1: Primer sequences descriptions of *VDR* polymorphisms; Table S2: Linkage disequilibrium between *VDR* polymorphisms in T1DM and non-T1DM groups; Table S3: Genotype frequency of rs7975232 and risk of type 1 diabetes; Table S4: Genotype frequency of rs731236 and risk of type 1 diabetes; Table S5: Genotype frequency of rs2228570 and risk of type 1 diabetes. Table S6: Haplotype test between *VDR* polymorphisms and T1DM.
