# Supplementary material for: Variants in the VDR Gene May Influence 25(OH)D Levels in Type 1 Diabetes Mellitus in a Brazilian Population
Source: Nutrients. 2022 Feb 27;14(5):1010. doi: 10.3390/nu14051010 (PMC8912721; doi:10.3390/nu14051010)
Supplement: Supplementary file 1 [file nutrients-14-01010-s001.zip › SUPPLEMENTARY TABLE S1.pdf]

Table S1. Primer sequences descriptions of *VDR* polymorphisms

Variant	Primers	Ta†
**rs7975232**	F: TCGGCTAGCTTCTGGATCAT<br>R: CTGCCGTTGAGTGTCTGTGT	57°C
**rs1544410**	F: CCATCTCTCAGGCTCCAAAG<br>R: CCTCACTGCCCTTAGCTCTG	56°C
**rs731236**	F: TCGGCTAGCTTCTGGATCAT<br>R: CTGCCGTTGAGTGTCTGTGT	57°C
**rs2228570**	F: TGCAGCCTTCACAGGTCATA<br>R: AGCTATGTAGGGCGAATCATGT	56°C

†Annealing temperature
