# Supplementary material for: Variants in the VDR Gene May Influence 25(OH)D Levels in Type 1 Diabetes Mellitus in a Brazilian Population
Source: Nutrients. 2022 Feb 27;14(5):1010. doi: 10.3390/nu14051010 (PMC8912721; doi:10.3390/nu14051010)
Supplement: Supplementary file 1 [file nutrients-14-01010-s001.zip › SUPPLEMENTARY TABLE S2 .pdf]

**Table S2.** Linkage disequilibrium between *VDR* polymorphisms in T1DM and non-T1DM groups.

| | **T1DM** | | **Non-T1DM** | |
|---|---|---|---|---|
| | **D´** | **r** | **D'** | **r** |
| **rs7975232 and rs731236** | 0.9995823 | -0.5105348 | 0.9993718 | -0.5632802 |
| **rs7975232 and rs1544410** | 0.9995580 | -0.5380158 | - | - |
| **rs7975232 and rs2228570** | 0.0865493 | -0.0434192 | 0.3013207 | 0.2364977 |
| **rs731236 and rs1544410** | 0.8021140 | 0.7611249 | - | - |
| **rs731236 and rs2228570** | 0.0587129 | 0.0576694 | 0.3194768 | -0.1413301 |
| **rs1544410 and rs2228570** | 0.0334862 | 0.0312103 | - | - |

Abbreviations: D', scaled linkage disequilibrium estimate; r, coefficient of correlation
