# Supplementary material for: Variants in the VDR Gene May Influence 25(OH)D Levels in Type 1 Diabetes Mellitus in a Brazilian Population
Source: Nutrients. 2022 Feb 27;14(5):1010. doi: 10.3390/nu14051010 (PMC8912721; doi:10.3390/nu14051010)
Supplement: Supplementary file 1 [file nutrients-14-01010-s001.zip › SUPPLEMENTARY TABLE S3.pdf]

Table S3. Genotype frequency of rs7975232 and risk of type 1 diabetes.

	Non-T1DM	%	T1DM	%	OR	lower	upper	*P-value*†	AIC
**Codominant**								0.5089	160.4
T/T	22	26.5	24	36.9	1				
G/T	48	57.8	35	53.8	0.71	0.29	1.76		
G/G	13	15.7	6	9.2	0.46	0.12	1.79		
**Dominant**								0.3426	158.9
T/T	22	26.5	24	36.9	1				
G/T-G/G	61	73.5	41	63.1	0.65	0.27	1.57		
**Recessive**								0.3685	159
T/T-G/T	70	84.3	59	90.8	1				
G/G	13	15.7	6	9.2	0.58	0.17	1.95		
**Overdominant**								0.7928	159.7
T/T-G/G	35	42.2	30	46.2	1				
G/T	48	57.8	35	53.8	0.9	0.4	2.02		
**log-Additive**								0.2468	158.4
0,1,2	83	56.1	65	43.9	0.69	0.36	1.3		

†Adjusted for age, sex, weight, body mass index, European ancestry and Native American ancestry.
Abbreviations: AIC, Akaike information criterion; OR, Odds Ratio; T1DM, type 1 diabetes mellitus.
