# Supplementary material for: Variants in the VDR Gene May Influence 25(OH)D Levels in Type 1 Diabetes Mellitus in a Brazilian Population
Source: Nutrients. 2022 Feb 27;14(5):1010. doi: 10.3390/nu14051010 (PMC8912721; doi:10.3390/nu14051010)
Supplement: Supplementary file 1 [file nutrients-14-01010-s001.zip › SUPPLEMENTARY TABLE S4.pdf]

Table S4. Genotype frequency of rs731236 and risk of type 1 diabetes.

	Non-T1DM	%	T1DM	%	OR	lower	upper	*P-value*†	AIC
**Codominant**								0.0866	156.9
T/T	40	48.2	32	49.2	1				
C/T	39	47	25	38.5	0.42	0.17	1.04		
C/C	4	4.8	8	12.3	1.61	0.3	8.48		
**Dominant**								0.1242	157.4
T/T	40	48.2	32	49.2	1				
C/T-C/C	43	51.8	33	50.8	0.51	0.22	1.22		
**Recessive**								0.2724	158.6
T/T-C/T	79	95.2	57	87.7	1				
C/C	4	4.8	8	12.3	2.39	0.5	11.46		
**Overdominant**								0.0323*	155.2
T/T-C/C	44	53	40	61.5	1				
C/T	39	47	25	38.5	0.39	0.16	0.95		
**log-Additive**								0.4568	159.2
0,1,2	83	56.1	65	43.9	0.78	0.39	1.52		

†Adjusted for age, sex, weight, body mass index, European ancestry and Native American ancestry.
Abbreviations: AIC, Akaike information criterion; OR, Odds Ratio; T1DM, type 1 diabetes mellitus.
