# Supplementary material for: Variants in the VDR Gene May Influence 25(OH)D Levels in Type 1 Diabetes Mellitus in a Brazilian Population
Source: Nutrients. 2022 Feb 27;14(5):1010. doi: 10.3390/nu14051010 (PMC8912721; doi:10.3390/nu14051010)
Supplement: Supplementary file 1 [file nutrients-14-01010-s001.zip › SUPPLEMENTARY TABLE S5.pdf]

Table S5. Genotype frequency of rs2228570 and risk of type 1 diabetes.

	Non-T1DM	%	T1DM	%	OR	lower	upper	*P-value*†	AIC
**Codominant**								0.9623	161.7
C/C	38	45.8	30	46.2	1				
T/C	35	42.2	30	46.2	1.13	0.48	2.66		
T/T	10	12	5	7.7	1.1	0.25	4.91		
**Dominant**								0.7833	159.7
C/C	38	45.8	30	46.2	1				
T/C-T/T	45	54.2	35	53.8	1.12	0.49	2.57		
**Recessive**								0.9651	159.8
C/C-T/C	73	88	60	92.3	1				
T/T	10	12	5	7.7	1.03	0.25	4.29		
**Overdominant**								0.8045	159.7
C/C-T/T	48	57.8	35	53.8	1				
T/C	35	42.2	30	46.2	1.11	0.49	2.51		
**log-Additive**								0.8153	159.7
0,1,2	83	56.1	65	43.9	1.08	0.57	2.05		

†Adjusted for age, sex, weight, body mass index, European ancestry and Native American ancestry.
Abbreviations: AIC, Akaike information criterion; OR, Odds Ratio; T1DM, type 1 diabetes mellitus.
