# Supplementary figures and images for: Variants in the VDR Gene May Influence 25(OH)D Levels in Type 1 Diabetes Mellitus in a Brazilian Population
Source: Nutrients. 2022 Feb 27;14(5):1010. doi: 10.3390/nu14051010 (PMC8912721; doi:10.3390/nu14051010)

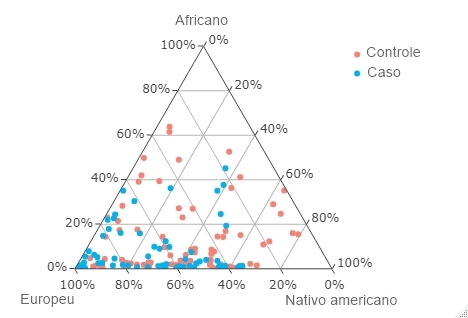

Supplement: Supplementary file 1 [file nutrients-14-01010-s001.zip › Supplementary Figure S1.jpeg]

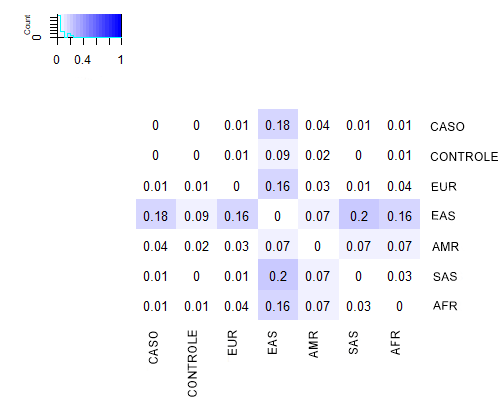

Supplement: Supplementary file 1 [file nutrients-14-01010-s001.zip › Supplementary Figure S2.tiff]

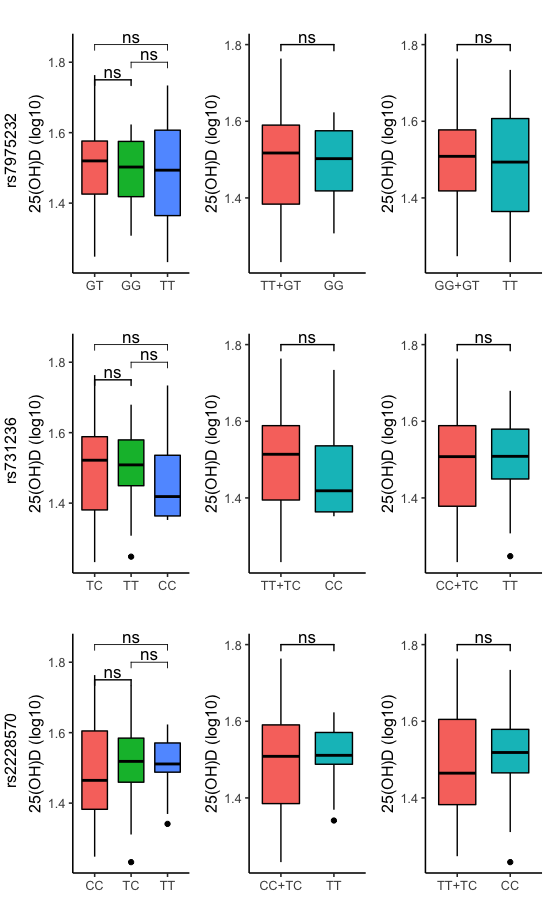

Supplement: Supplementary file 1 [file nutrients-14-01010-s001.zip › Supplementary Figure S3.tiff]
